# Association between Inflammation-Based Parameters and Prognosis in Patients with Acute Kidney Injury

**DOI:** 10.3390/medicina57090936

**Published:** 2021-09-05

**Authors:** Hyelim Joo, Sun Young Min, Min-Su Park

**Affiliations:** Department of Surgery, School of Medicine, Kyung Hee University, Seoul 02447, Korea; kaylin-j@daum.net (H.J.); minishan@naver.com (S.Y.M.)

**Keywords:** acute kidney injury, inflammation, prognostic factor

## Abstract

*Background and Objectives*: this study aimed to clarify the relationship between inflammation-based parameters and prognosis in patients with acute kidney injury (AKI). *Materials and Methods*: We analyzed the prospectively collected data of patients with AKI, who were admitted through the emergency department between March 2020 and April 2021. Their clinical characteristics, inflammation-based parameters, resolving/non-resolving AKI pattern, and major adverse kidney event (MAKE) rates were analyzed. *Results*: Among 177 patients, 129 (72.9%) had a resolving AKI pattern and 48 (27.1%) had a non-resolving AKI pattern. The outcome of MAKE occurred in 30 (16.9%) participants. Multivariate analyses showed that the neutrophil-to-monocyte ratio was an independent predictor of resolving AKI, and that the neutrophil-to-monocyte and neutrophil-to-lymphocyte ratios were independent predictors of MAKE occurrence. *Conclusions*: we demonstrated that inflammation-based parameters are valuable predictors of early recovery and MAKE occurrence in patients with AKI.

## 1. Introduction

Acute kidney injury (AKI) is a pathological condition that impairs the structure and function of the kidney, and is characterized by a sudden impairment of the functions of the kidney. The risk factors and renal susceptibility to acute injury have been widely studied. AKI is a broad clinical syndrome with complex and diverse causes, such as secondary damage, caused by nephrotoxic drugs, infection, hypovolemia, and urinary obstruction [1,2,3].

The overall incidence of AKI has recently increased, ranging from 5% to 25%, and is associated with high morbidity and mortality. In addition, AKI increases the risk of chronic kidney disease (CKD) and end-stage renal disease [2,4,5]. In AKI, it is important to initially identify the causes of renal damage and to orient the treatment. It is also important to infer AKI recovery patterns, based on various causes, risks, and blood test results of patients, and to predict severe complications, such as long-term dialysis and chronic kidney disease. Therefore, the discovery of prognostic markers of AKI that are easy to assess in clinical practice can assist decision making in the management of patients with AKI.

The increased frequency of the systemic inflammatory response plays important roles in the development of AKI. Several studies have focused on the importance of the systemic inflammatory status in predicting the occurrence of AKI. Many studies demonstrated that the values for several inflammation-based parameters, such as the neutrophil-to-lymphocyte ratio (NLR), platelet-to-lymphocyte ratio (PLR), albumin-to-globulin ratio (AGR), and prognostic nutritional index (PNI), have been reported to correlate with the development of AKI [6,7,8,9,10].

However, the usefulness of inflammation-based parameters in assessing patients with AKI has not yet been determined in terms of prognosis. Few studies have evaluated the relationship between inflammation-based markers and clinical outcomes in patients with AKI. Therefore, in this study, we aimed to clarify the relationship between inflammation-based parameters and prognosis in patients with AKI.

## 2. Materials and Methods

### 2.1. Patients

We analyzed the prospectively collected data of 204 patients with AKI who were admitted through the emergency department at Kyunghee University Hospital, Korea, between March 2020 and April 2021. AKI was defined using modified KDIGO criteria based on an increase in serum creatinine concentration of ≥50% or ≥0.3 mg/dL above an outpatient, non-emergency department baseline value, within 7–365 days before admission [11]. Urine output-based criteria were not included in the definition of AKI because they were not reliably recorded in the emergency department. The exclusion criteria were as follows: loss to follow-up, lack of routine blood indexes, and transfer to another hospital. Finally, 177 AKI patients who had ended follow-up were included in this study.

### 2.2. Definition of Clinical Outcome

The staging of AKI is defined as follows: stage 1, serum creatinine level increased by ≥0.3 mg/dL (≥26 mmol/L) within 48 h or increased by 1.5–1.9 times from the baseline value; stage 2, serum creatinine level increased by 2.0–2.9 times from the baseline value; stage 3, serum creatinine level increased by ≥4.0 mg/dL (≥354 mmol/L) within 48 h or increased by ≥3.0 times from the baseline value or the requirement of renal replacement therapy by the patient [11]. Resolving AKI was defined as a decrease in serum creatinine concentration of ≥0.3 mg/dL or ≥25% from the maximum value in the first 72 h after AKI diagnosis. Non-resolving AKI was defined as not meeting the definition of resolving AKI. Major adverse kidney events (MAKEs) were defined as incident or progressive CKD, long-term dialysis, or all-cause mortality during admission [12,13].

### 2.3. Evaluation of Systemic Inflammatory and Nutritional Indexes

NLR was calculated by dividing the neutrophil count by the lymphocyte count. The PLR was calculated by dividing the platelet count by the lymphocyte count. The lymphocyte-to-monocyte ratio (LMR) was calculated by dividing the lymphocyte count by the monocyte count. The neutrophil-to-monocyte ratio (NMR) was calculated by dividing the neutrophil count by the monocyte count. The PNI was calculated as 10 × serum albumin level + 0.005 × total peripheral lymphocyte count (per mm3). The C-reactive protein-to-albumin ratio (CAR) was calculated by dividing the serum C-reactive protein (CRP) level by the serum albumin level. The modified Glasgow prognostic score was calculated as follows: GPS 2, both CRP >1.0 mg/dL and albumin <3.5 g/dL; GPS 1, either CRP >1.0 mg/dL or albumin <3.5 g/dL, but not both; and GPS 0, neither abnormality. AGR was calculated using the following equation: albumin/(total protein-albumin).

### 2.4. Statistical Analysis

The associations of continuous and categorical variables with the relevant outcome variables were assessed using Student’s t-test and chi-square test, respectively. Data are presented as mean ± standard deviation. To investigate the diagnostic performance of inflammation-based parameters in the clinical prognosis of patients with AKI, receiver operating characteristic (ROC) analysis was performed. We conducted a multivariate logistic regression analysis to identify the risk factors associated with the clinical prognosis of patients with AKI. Forward stepwise selection using likelihood ratios for entry and exit criteria was used to develop the final multivariate logistic regression analysis model. A *p* value < 0.05 was considered statistically significant.

## 3. Results

### 3.1. Patient Characteristics

We enrolled 177 patients (96 male, 54.2%; mean age, 71.6 ± 14.8 years), of whom 129 (72.9%) had a resolving AKI pattern and 48 (27.1%) had a non-resolving AKI pattern. The outcome of a MAKE occurred in 30 (16.9%) participants. The patients’ baseline eGFR was 19.6 ± 13.4 mL/min/1.73 m2 in the resolving AKI group and 21.1 ± 16.5 mL/min/1.73 m^2^ in the non-resolving AKI group. No significant differences were found for inflammation-based parameters between the two groups. Non-resolving AKI was associated with a greater risk of MAKE occurrence (Table 1).

### 3.2. Cut-Off Values for Inflammation-Based Markers

The areas under the curve, cut-off values, and sensitivities and specificities of inflammation-based markers, based on the results of time-dependent ROC curve analyses, are shown in Table 2. According to the cut-off value of 3.43 for CRP, 12.71 for NLR, 47.12 for PLR, 885.84 for PNI, 5.38 for LMR, 13.28 for NMR, 2.85 for CAR, and 1.31 for AGR, the patients were divided into high and low groups (Table 2).

### 3.3. Association of Inflammation-Based Parameters with AKI Recovery Patterns

Univariate analysis showed that NMR (*p* = 0.018) and NLR (*p* = 0.021) were independent factors associated with early AKI recovery. Multivariate analysis showed NMR to be an independent prognostic factor for resolving AKI (hazard ratio [HR], 2.451; 95% confidence interval [CI], 1.170–5.135; *p* = 0.018) (Table 3).

### 3.4. Association of Inflammation-Based Parameters with MAKEs

Univariate analysis showed that NMR (*p* = 0.003), NLR (*p* = 0.004), and the KDIGO stage (*p* = 0.005) were independent factors associated with MAKE occurrence. Multivariate analysis showed NMR (HR, 4.267; 95% CI, 1.385–13.147; *p* = 0.012) and NLR (HR, 2.472; 95% CI, 1.072–5.702; *p* = 0.034) to be independent prognostic factors for MAKE occurrence (Table 4).

## 4. Discussion

The prognosis of acute kidney injury is determined by a prompt response, according to the cause. In addition, it is essential to predict the patient’s risk according to the patient’s condition. For patients visiting the emergency room, it is important to predict the recovery pattern of renal function and the occurrence of severe complications through inflammation-based parameters that are relatively easy and fast to perform. In this study, multivariate NMR was shown to be an independent predictor of resolving AKI, and NMR and NLR were shown to be independent predictors of MAKE occurrence. This demonstrates that inflammation-based parameters are valuable predictors for early recovery and MAKE occurrence in patients with AKI.

Rabb et al. demonstrated that AKI leads to a series of events in which numerous inflammatory factors and inflammatory cells promote oxidative stress and apoptosis, eventually leading to renal failure [14]. Prompt treatment that is appropriate to the etiology of AKI is important, although it is also important to restore kidney function and re-evaluate the patient’s prognosis after acute injury. Studies of prognostic stratification in patients have recently been reported, focusing on the importance of AKI recovery patterns and the role of protective strategies. Kelluem et al. linked specific phenotypes and recovery patterns to the outcomes of patients with AKI in their study [15]. Bhatraju et al. demonstrated that the risk of clinically significant kidney-specific long-term outcomes, such as MAKE, could be determined within 72 h of AKI. The identification of various AKI recovery patterns can improve patient risk stratification and promote the prognosis [16].

Numerous studies have attempted to confirm the usefulness of inflammation-based parameters in the development of AKI. However, there have been few studies on the effects of inflammation-based parameters on recovery patterns and long-term clinical outcomes in patients with AKI. In addition, optimal indexes of inflammation-based parameters for recovery in patients with AKI have not yet been established.

NMR is a systemic blood marker of inflammation that reflects the progression of various diseases. Several studies have demonstrated that NMR can predict the outcomes of various diseases, including CKD and malignant disease [17,18]. Our study showed that NMR at admission was an independent prognostic factor for the early recovery of AKI, and patients with NMR ≥ 13.28 showed significantly lower incidences of resolving AKI. The high tendency of inflammation in the NMR ≥ 13.28 group may be a reason why NMR was shown to be an independent predictor to MAKE occurrence in patients with AKI.

NLR is an indicator that reflects a patient’s nutritional and immune status. A previous study revealed that NLR is a predictor of AKI occurrence and severe AKI in patients undergoing gastrointestinal and hepatobiliary surgery in the ICU [9]. Our study showed that NLR at admission was an independent predictor of MAKE occurrence, and patients with NLR ≥ 12.71 showed significantly higher incidences of MAKEs.

In the previous analysis, various risk factors that were related to the prognosis of AKI included the condition of the causative disease, age, severity of the disease and co-morbid disease. However, this study did not show significant results about these variables. Perhaps this is because the target patient is different and various causes are related compared to other studies.

Our study had some limitations. First, this was a single-center cohort study. Second, we retrospectively analyzed the electronic medical records of only a few patients. The small sample size and retrospective single-center design may have limited the findings’ generalizability. A multiple-center study, using a larger sample size, is needed to clarify the relationship between inflammation-based parameters and prognosis in patients with AKI. A high incidence of AKI has been reported in association with COVID-19 infection, possibly due to underlying inflammation mediated by cytokines. In addition, it has been reported that uncovered AKI appears in a high proportion [19]. Further studies on the association between inflammation-based parameters and AKI in the COVID-19 setting are needed in the future.

## 5. Conclusions

We showed that NMR and NLR might represent novel and useful inflammatory prognostic scores for patients with AKI. NMR was an independent predictor of resolving AKI and MAKE occurrence. NLR was an independent predictor of MAKE occurrence. Inflammation-based parameters might be valuable predictors for early recovery and MAKE occurrence in patients with AKI. For the rapid recovery of renal function in patients with AKI, it is important to perform systemic inflammatory and nutritional index tests in the emergency room, and to analyze the results to predict the risk and promptly determine the direction of treatment. If these tests are performed in the emergency room as soon as possible, it will be helpful in determining the prognosis for the patient’s hospitalization period and renal function recovery, taking into account the underlying and merged acute diseases.

## Figures and Tables

**Table 1:** Demographic and clinical features of patients with acute kidney injury.

Variable	Overall (*n*= 177)	Resolving AKI (*n* = 129)	Non-Resolving AKI (*n* = 48)	*p* Value ^a^
Age (y)	71.68 ± 14.85	72.2 ± 14.5	70.1 ± 15.5	0.390
Sex (Male)	96 (54.2)	70 (54.3)	26 (54.2)	0.991
Diabetes mellitus (%)	83 (46.9)	60 (46.5)	23 (47.9)	0.823
Hypertension (%)	114 (64.4)	83 (64.3)	31 (64.6)	0.976
Inflammatory disease (%)	24 (13.6)	15 (11.6)	9 (18.8)	0.219
Chronic kidney disease (%)	77 (43.5)	49 (38.0)	28 (58.3)	0.015
Cerebrovascular accident (%)	42 (23.7)	27 (20.9)	15 (31.3)	0.151
Malignancy (%)	32 (18.1)	23 (17.8)	9 (18.8)	0.887
Heart failure (%)	31 (17.5)	21 (16.3)	10 (20.8)	0.479
Presumed main cause				0.505
Hypovolemia (%)	157 (88.7)	116 (89.9)	41 (85.4)	
Infection (%)	11 (6.2)	6 (4.6)	5 (10.4)	
Heart failure (%)	6 (3.4)	5 (3.9)	1 (2.1)	
Medication (%)	3 (1.7)	2 (1.6)	1 (2.1)	
KDIGO stage of AKI (%)				0.124
1	92 (52.0)	69 (53.5)	23 (47.9)	
2	22 (12.4)	19 (14.7)	3 (6.3)	
3	63 (35.6)	41 (31.8)	22 (45.8)	
BUN (mg/dL)	66.78 ± 42.39	66.1 ± 40.5	68.5 ± 47.3	0.739
Creatinine (mg/dL)	4.1 ± 3.0	4.0 ± 2.4	4.3 ± 4.2	0.507
eGFR (mL/min/1.73 m^2^)	20.0 ± 14.3	19.6 ± 13.4	21.1 ± 16.5	0.518
CRP	7.1 ± 9.2	6.5 ± 8.5	8.7 ± 10.8	0.210
NLR	11.5 ± 13.1	10.7 ± 11.9	13.7 ± 16.0	0.237
PLR	28.9 ± 28.9	27.4 ± 26.6	33.0 ± 34.4	0.254
PNI	651.6 ± 412.4	664.9 ± 420.2	616.0 ± 392.8	0.485
mGPS				0.070
0	126 (71.2)	95 (73.6)	31 (64.6)	
1	33 (18.6)	19 (14.7)	14 (29.2)	
2	18 (10.2)	15 (11.6)	3 (6.3)	
LMR	2.8 ± 2.0	2.7 ± 1.9	2.9 ± 2.2	0.685
NMR	19.0 ± 13.3	17.7 ± 10.3	22.5 ± 18.8	0.107
CAR	2.3 ± 3.2	2.0 ± 2.8	3.1 ± 4.1	0.096
AGR	1.1 ± 1.0	1.1 ± 1.1	1.0 ± 0.6	0.548
Mechanical ventilation (%)	7 (4.0)	3 (2.3)	4 (8.3)	0.068
Dialysis (%)	21 (11.9)	8 (6.2)	13 (27.1)	<0.001
MAKE (%)	30 (16.9)	9 (7.0)	21 (43.8)	<0.001

^a^ Comparing the outcome of AKI recovery between patients with resolving AKI and patients with non-resolving AKI. AKI, acute kidney injury; AGR, albumin–globulin ratio; CRP, C-reactive protein; CAR, C-reactive protein-to-albumin ratio; LMR, lymphocyte-to-monocyte ratio; mGPS = modified Glasgow prognostic score, NLR, neutrophil-to-lymphocyte ratio; NMR, neutrophil-to-monocyte ratio; PLR, platelet-to-lymphocyte ratio; PNI, prognostic nutritional index.

**Table 2:** Discrimination ability and cut-off values for inflammation-based markers.

Variable	AUC	Cut-Off	Sensitivity (%)	Specificity (%)
CRP	0.532	3.43	52	60
NLR	0.555	12.71	45	74
PLR	0.547	47.12	29	82
PNI	0.463	885.84	25	81
LMR	0.494	5.38	18	90
NMR	0.574	13.28	77	45
CAR	0.553	2.85	37	75
AGR	0.451	1.31	37	67

AGR, albumin–globulin ratio; CRP, C-reactive protein; CAR, C-reactive protein-to-albumin ratio; LMR, lymphocyte-to-monocyte ratio; NLR, neutrophil-to-lymphocyte ratio; NMR, neutrophil-to-monocyte ratio; PLR, platelet-to-lymphocyte ratio; PNI, prognostic nutritional index.

**Table 3:** Association of inflammation-based parameters with acute kidney injury recovery patterns.

Variable	Category	Hazard Ratio (95% CI)	*p* Value
NMR	Low (≤13.28)	Reference	
	High (>13.28)	2.451 (1.170–5.135)	0.018

NMR, neutrophil-to-monocyte ratio.

**Table 4:** Association of inflammation-based parameters with major adverse kidney events.

Variable	Category	Hazard Ratio (95% CI)	*p* Value
NMR	Low (≤13.28)	Reference	
	High (>13.28)	4.267 (1.385–13.147)	0.012
NLR	Low (≤12.71)	Reference	
	High (>12.71)	2.472 (1.072–5.702)	0.034

NLR, neutrophil-to-lymphocyte ratio; NMR, neutrophil-to-monocyte ratio.

## Data Availability

Not applicable.

## References

[1] Murray P.T., Mehta R.L., Shaw A., Ronco C., Endre Z., Kellum J.A., Chawla L.S., Cruz D., Ince C., Okusa M.D. (2014). Potential use of biomarkers in acute kidney injury: Report and summary of recommendations from the 10th Acute Dialysis Quality Initiative consensus conference. Kidney Int..

[2] Ronco C. (2016). Acute kidney injury: From clinical to molecular diagnosis. Crit. Care.

[3] Ronco C., Ferrari F., Ricci Z. (2017). Recovery after Acute Kidney Injury: A New Prognostic Dimension of the Syndrome. Am. J. Respir. Crit. Care Med..

[4] Fang Y., Ding X., Zhong Y., Zou J., Teng J., Tang Y., Lin J., Lin P. (2010). Acute kidney injury in a Chinese hospitalized population. Blood Purif..

[5] Lafrance J.P., Miller D.R. (2010). Acute kidney injury associates with increased long-term mortality. J. Am. Soc. Nephrol. JASN.

[6] Owhin S.O., Abejegah C., Fasipe O.J., Oke C., Abidoye A., Osagbaekhoe A., Awe A., Etafo I., Iredia E., Ayodeji O. (2020). Association of hypoalbuminemia and reversal of albumin-to-globulin ratio with morbidity outcome among hospitalized Lassa fever infected patients at a dedicated treatment center in Ondo state, south-western Nigeria. Future Sci. OA.

[7] Sim J.H., Bang J.Y., Kim S.H., Kang S.J., Song J.G. (2021). Association of Preoperative Prognostic Nutritional Index and Postoperative Acute Kidney Injury in Patients with Colorectal Cancer Surgery. Nutrients.

[8] Chen D., Xiao D., Guo J., Chahan B., Wang Z. (2020). Neutrophil-lymphocyte count ratio as a diagnostic marker for acute kidney injury: A systematic review and meta-analysis. Clin. Exp. Nephrol..

[9] Bi J.B., Zhang J., Ren Y.F., Du Z.Q., Wu Z., Lv Y., Wu R.Q. (2020). Neutrophil-to-lymphocyte ratio predicts acute kidney injury occurrence after gastrointestinal and hepatobiliary surgery. World J. Gastrointest. Surg..

[10] Dolapoglu A., Avci E., Kiris T., Bugra O. (2019). The predictive value of the prognostic nutritional index for postoperative acute kidney injury in patients undergoing on-pump coronary bypass surgery. J. Cardiothorac. Surg..

[11] Fliser D., Laville M., Covic A., Fouque D., Vanholder R., Juillard L., van Biesen W., Ad-hoc working group of ERBP (2012). A European Renal Best Practice (ERBP) position statement on the Kidney Disease Improving Global Outcomes (KDIGO) clinical practice guidelines on acute kidney injury: Part 1: Definitions, conservative management and contrast-induced nephropathy. Nephrol. Dial. Transplant. Off. Publ. Eur. Dial. Transpl. Assoc.-Eur. Ren. Assoc..

[12] Bhatraju P.K., Mukherjee P., Robinson-Cohen C., O’Keefe G.E., Frank A.J., Christie J.D., Meyer N.J., Liu K.D., Matthay M.A., Calfee C.S. (2016). Acute kidney injury subphenotypes based on creatinine trajectory identifies patients at increased risk of death. Crit. Care.

[13] McKown A.C., Wang L., Wanderer J.P., Ehrenfeld J., Rice T.W., Bernard G.R., Semler M.W. (2017). Predicting Major Adverse Kidney Events among Critically Ill Adults Using the Electronic Health Record. J. Med. Syst..

[14] Rabb H., Griffin M.D., McKay D.B., Swaminathan S., Pickkers P., Rosner M.H., Kellum J.A., Ronco C. (2016). Acute Dialysis Quality Initiative Consensus, X.W.G. Inflammation in AKI: Current Understanding, Key Questions, and Knowledge Gaps. J. Am. Soc. Nephrol. JASN.

[15] Kellum J.A., Sileanu F.E., Bihorac A., Hoste E.A., Chawla L.S. (2017). Recovery after Acute Kidney Injury. Am. J. Respir. Crit. Care Med..

[16] Bhatraju P.K., Zelnick L.R., Chinchilli V.M., Moledina D.G., Coca S.G., Parikh C.R., Garg A.X., Hsu C.Y., Go A.S., Liu K.D. (2020). Association Between Early Recovery of Kidney Function After Acute Kidney Injury and Long-term Clinical Outcomes. JAMA Netw. Open.

[17] Davila-Collado R., Jarquin-Duran O., Solis-Vallejo A., Nguyen M.A., Espinoza J.L. (2021). Elevated Monocyte to Lymphocyte Ratio and Increased Mortality among Patients with Chronic Kidney Disease Hospitalized for COVID-19. J. Pers. Med..

[18] Panni R.Z., Lopez-Aguiar A.G., Liu J., Poultsides G.A., Rocha F.G., Hawkins W.G., Strasberg S.M., Trikalinos N.A., Maithel S., Fields R.C. (2019). Association of preoperative monocyte-to-lymphocyte and neutrophil-to-lymphocyte ratio with recurrence-free and overall survival after resection of pancreatic neuroendocrine tumors (US-NETSG). J. Surg. Oncol..

[19] Ng J.H., Hirsch J.S., Hazzan A., Wanchoo R., Shah H.H., Malieckal D.A., Ross D.W., Sharma P., Sakhiya V., Fishbane S. (2021). Outcomes among patients hospitalized with COVID-19 and acute kidney injury. Am. J. Kidney Dis..

